# Isocudraxanthone K Induces Growth Inhibition and Apoptosis in Oral Cancer Cells via Hypoxia Inducible Factor-1***α***

**DOI:** 10.1155/2014/934691

**Published:** 2014-07-03

**Authors:** Mee-Ran Shin, Hwa-Jeong Lee, Soo-Kyung Kang, Q-Schick Auh, Young-Man Lee, Youn-Chul Kim, Eun-Cheol Kim

**Affiliations:** ^1^Department of Prosthodontics, Dongtan Sacred Heart Hospital, Hallym University, Dongtan, Republic of Korea; ^2^Department of Oral and Maxillofacial Pathology, and Research Center for Tooth and Periodontal Regeneration (MRC), School of Dentistry, Kyung Hee University, 1 Heogi-dong, Dongdaemun-gu, Seoul 130-701, Republic of Korea; ^3^Department of Oral Medicine, School of Dentistry, Kyung Hee University, Seoul, Republic of Korea; ^4^College of Pharmacy, Wonkwang University, Iksan 570-749, Republic of Korea

## Abstract

Isocudraxanthone K (IK) is a novel, natural compound from a methanol extract of the root bark of * Cudrania tricuspidata*. It has not been shown previously that IK possessed antitumor activity. We investigated the antitumor effects and molecular mechanism of IK and related signal transduction pathway(s) in oral squamous cell carcinoma cells (OSCCCs). The MTT assay revealed that IK had an antiproliferative effect on OSCCCs, in a dose- and time-dependent manner. IK induced apoptosis in OSCCCs, as identified by a cell-cycle analysis, annexin V-FITC and propidium iodide staining, and the nuclear morphology in cell death. IK caused time-dependent phosphorylation of Akt, p38, and ERK (extracellular signal-regulated kinase). In addition, IK increased the cytosolic to nuclear translocation of nuclear factor-*κ*B (NF-*κ*B) p65 and the degradation and phosphorylation of I*κ*B-*α* in HN4 and HN12 cells. Furthermore, IK treatment downregulated hypoxia-inducible factor 1*α* (HIF-1*α*) and its target gene, vascular endothelial growth factor (VEGF). Cobalt chloride (CoCl_2_), a HIF-1*α* activator, attenuated the IK-induced growth-inhibiting and apoptosis-inducing effects, and blocked IK-induced expression of apoptosis regulatory proteins, such as Bax, Bcl-2, caspase-3, caspase-8, and caspase-9, and cytochrome c. Collectively, these data provide the first evidence of antiproliferative and apoptosis-inducing effects of IK as a HIF-1*α* inhibitor and suggest it may be a drug candidate for chemotherapy against oral cancer.

## 1. Introduction

Oral squamous cell carcinoma accounts for more than 90% of oral cancers [1] and its prognosis remains dismal; indeed, more than 50% of patients die of the disease or complications within 5 years under current therapies [2]. Treatment of oral cancer has relied primarily on the “classical” modalities of surgery, radiation, and chemotherapy and combinations of these methods [3]. Conventional chemotherapeutic agents have been associated with numerous significant clinical complications, including nausea, hair loss, and pancytopenia. Thus, there is a continuing need for alternative and less toxic treatments for oral cancers [4].

One approach for developing clinically effective chemotherapeutic agents is to screen natural products that have been used in traditional medicine with few side effects for anticancer activities [5]. Recently, the targeted elimination of OSCCCs by inducing apoptosis, such as by targeting Bcl-2, cFLIP (caspase-8-inhibitory protein, an inhibitor of Fas activation), inhibitors of AKT (an antiapoptotic serine-threonine kinase), and nuclear factor-*κ*B (NF-*κ*B), has emerged as a valuable strategy to combat oral cancer [6–8]. Previously, we demonstrated that herbal medicine ingredients, such as* Caesalpinia sappan* [9],* Coptidis rhizome* [10], verticinone [11], sappanchalcone [12], isoliquiritigenin 2′-methyl ether [13], and mollugin [14], had antitumor effects on oral cancer cells* in vitro*.

Hypoxia-inducible factor-1 (HIF-1) is a transcription factor that plays an essential role in oxygen homeostasis; it is a heterodimer composed of HIF-1*α* and HIF-1*β* [15]. The HIF-1*α* protein is degraded rapidly under normoxic conditions and stabilized under hypoxic conditions, while HIF-1*β* protein is expressed constitutively. HIF-1*α* has been recognized as an important regulatory protein in the transcription of many genes related to glucose transport, glycolysis, erythropoiesis, cell proliferation/survival, and angiogenesis [16].

Overexpression of the HIF-1*α* subunit has been observed in many human cancers and increased levels of HIF-1*α* protein correlate with advanced disease stages and poor prognosis [17]. Inhibition of HIF-1*α* function in tumors has been shown to attenuate or suppress tumor growth in experimental xenograft models [17, 18]. Thus, small-molecule HIF-1*α* inhibitors from natural products have potential as molecularly targeted cancer therapeutics [15, 16]. Indeed, HIF-1 inhibitors for cancer therapy, such as apigenin [19, 20], laurenditerpenol [21], and emetine analogs [22], have been reported from natural sources.

*Cudrania tricuspidata* (*C. tricuspidata*) is used in traditional herbal remedies for inflammation, gastritis, tumors, and hepatitis in Korea, China, and Japan [23]. Previous studies have shown that the constituents of* C. tricuspidata* extract have antioxidant [24, 25], antiatherosclerotic, anti-inflammatory [26], and anticancer effects in leukemia HL-60 cells [27], colon carcinoma [28], hepatocellular carcinoma [28], gastric carcinoma [28], U937, and HeLa cells [29]. We reported previously that compounds isolated from* C. tricuspidata* roots exhibited hepatoprotective effects [30]. Furthermore, we demonstrated that cudratricusxanthone A, isolated from the roots of* C. tricuspidata*, was able to protect mouse hippocampal cells against glutamate-induced neurotoxicity via induction of heme oxygenase-1 (HO-1) [31]. Additionally, we recently demonstrated that cudraflavone B, isolated from the root bark of* C. tricuspidata*, has antitumor effects in OSCCCs via MAPK and NF-*κ*B and a SIRT1 pathway [32].

We isolated isocudraxanthone K (IK) previously, a natural compound from the CHCl_3_-soluble fraction of a methanol extract of the root bark of* C. tricuspidata* [33]. However, anticancer effects of IK have not been reported before. The purpose of this study was to investigate the effects of IK, isolated from* C. tricuspidata*, on the growth of and the induction of apoptosis in human primary and metastatic OSCCCs and to identify the signaling pathway(s) involved.

## 2. Materials and Methods

### 2.1. Preparation of Isocudraxanthone K

Isocudraxanthone K (>97% pure) was isolated from the roots of* Cudrania tricuspidata*, as described by An et al. [33]. Root bark of* C. tricuspidata *was purchased in June 2004 at the Kumsan crudedrug market, Chungnam Province, Korea, and was identified by Dr. Kyu-Kwan Jang of the botanical gardens at Wonkwang University. A voucher specimen (No. WP 527) was deposited at the Herbarium of the College of Pharmacy, Wonkwang University (Korea). Isocudraxanthone K (NNMBP032) was deposited at the New Natural Material Bank of the College of Pharmacy, Wonkwang University, Korea. The identity of the isolated IK was confirmed by comparing the measured data obtained from UV, [*α*]_*D*_, MS, ^1^H, and ^13^C NMR spectroscopy [33].

### 2.2. Cell Culture

Human keratinocyte HaCaT cells, a nontransformed human cell line, were incubated in DMEM supplemented with 10% FBS, 1 mM sodium pyruvate, 50 *μ*g/mL streptomycin, and 50 *μ*g/mL penicillin at 37°C in 5% CO_2_.

The cell line HNSCC4 (HN4) from a primary T_3_N_0 _M_0_ carcinoma of the mouth floor and cell line HNSCC12 (HN12), from a metastatic carcinoma of the oral cavity [34], were derived in the laboratory of Dr. John F. Ensley (Wayne State University). All cell lines were grown at 37°C in a humidified 5% CO_2_ + 95% air atmosphere. Cells were dissociated with 0.25% trypsin immediately before transferring for experiments and were counted using a hemocytometer.

### 2.3. Cytotoxicity Assay

To evaluate the cytotoxicity of IK, the MTT colorimetric assay was performed to determine cell viability. Briefly, cells were seeded in flat-bottomed 96-well plates, at 1 × 10^5^ cells/well 24 h prior to treatment. The cells were treated for various time periods with the agents indicated. Then 25 *μ*L of 5 mg/mL MTT were added to each well. After a 4-hour incubation at 37°C, 100 *μ*L of lysing buffer (20% w/v sodium dodecyl sulfate in 0.1% HCl solution) was added. The plates were read with an ELISA reader at 570 nm.

### 2.4. Flow Cytometric Analysis

To determine the effects of IK on the cell cycle, cells were exposed to IK for 2 days. Then, cells were washed, fixed with 70% ethanol, and incubated for 30 min at 37°C with 0.1% RNAse A in PBS. Cells were then washed again, resuspended, and stained in PBS containing 25 *μ*g/mL propidium iodide (PI) for 30 min at room temperature. The cell distribution across the cell cycle was analyzed using a FACSCalibur (Becton Dickinson, Bedford, MA).

FITC-annexin V/propidium iodide (PI) double staining was also performed. After washing twice with PBS, cells (1 × 10^6^) were resuspended in binding buffer (10 mM HEPES/NaOH, pH 7.4, 140 mM NaCl, and 2.5 mM CaCl_2_) and FITC-annexin V and PI, at concentrations of 1 *μ*g/mL each, were added. The mixture was incubated for 10 min in the dark at room temperature and then cellular fluorescence was measured by flow cytometry analysis.

### 2.5. Fluorescent Staining of Nuclei with DAPI

To visualize chromatin condensation, cells (1 × 10^5^) were fixed in 4% paraformaldehyde, and DNA was stained with DAPI (1 *μ*g/mL; Sigma). The condensation state of the chromatin was visualized by fluorescence microscopy (Olympus, Japan).

### 2.6. Western Blot Analysis

Cells where harvested by centrifugation, washed twice in PBS, resuspended in icecold lysis buffer (1% Triton X-100, 45 mM KCl, 10 mM Tris, pH 7.5), supplemented with protease and phosphatase inhibitors, and then subjected to SDS-PAGE in 10% or 12% polyacrylamide gels, followed by protein transfer to a Hybond-P membrane (Amersham Pharmacia Biotech, Little Chalfont, UK), and treated with blocking solution for 1 h and incubated with primary antibody for phospho-ERK (p-ERK, cell signaling, Beverly, MA), ERK (cell signaling), p-JNK (cell signaling), JNK (cell signaling), p-I*κ*B (Santa Cruz Biotechnology, Santa Cruz, CA), I*κ*B (Santa Cruz Biotechnology), p65 (Santa Cruz Biotechnology), phospho-Akt (Santa Cruz Biotechnology), Akt (Santa Cruz Biotechnology), caspase family (Santa Cruz Biotechnology), Bcl-2 (Santa Cruz Biotechnology), Bax (Santa Cruz Biotechnology), cytochrome c (Santa Cruz Biotechnology), and *β*-actin (Sigma-Aldrich, St. Louis, MO). Following three washes with PBS-T, membranes were incubated in secondary antibody (1 : 5000, anti-mouse IgG-HRP labelled, R&D Systems, Minneapolis, MN) for 1 h. Signal was detected using an enhanced chemiluminescence system (Amersham-Pharmacia, Piscataway, NJ) according to the manufacturer's instructions. Densitometric analysis of each blot was performed with a computerized image processing system (Quantity One; Bio-Rad, Hercules, CA).

### 2.7. Reverse Transcriptase-PCR (RT-PCR)

Total RNA was extracted with TRIzol (BRL Life and Technologies, MD). cDNAs were prepared and amplified from 2 *μ*g of total RNA using the ThermoScript RT-PCR system with oligo (dT)_12-18_ (Invitrogen, Carlsbad, CA), analyzed on 2% agarose gels, and confirmed by nucleotide sequencing. The following primer pairs were used for RT-PCR: HIF-1*α* (325 bp): 5′-ACTTCTGGATGCTGGTGATT-3′ (sense) and 5′-TCCTCGGCTAGTTAG GGTAC-3′ (antisense), HIF-1*β* (264 bp): 5′-ATGTCTAACGATAAGGAGCGGTTT-3′ (sense) and 5′-AAGTTTATCCACATCATCTGGGTG-3′ (antisense), cyclin A1 (415 bp): 5′-GCCTGGCAAACTATACTGTG-3′ (sense) and 5′-CTCCATGAGGGACACACACA-3′ (anti-sense), cyclin D1 (726 bp): 5′-CCCTCGGTGTCCTACTTCAAA-3′ (sense) and 5′-CACCTCCTCCTCCTCCTCTTC-3′ (antisense), and *β*-actin: 5′-GTTGCGTTACACCCTTTCTTG-3′ (sense) and 5′-TGCTGTCACCTTCACCGT TC-3′ (antisense, the amplimer was 133 bp). Amplification conditions were 95°C for 3 min, 35 cycles of denaturation at 95°C for 30 s, annealing at 55°C for 30 s, and extension at 72°C for 30 s. PCR products were detected using agarose gel electrophoresis. Densitometric analysisof PCR bandswas performed with a computerized image processing system (Quantity One; Bio-Rad, Hercules, CA).

### 2.8. Cell Migration Assay

Cell migration assays used Costar Transwell System (8-*μ*m pore size polycarbonate membrane, 6.5-mm diameter, Corning, Inc., Corning, NY) following manufacturer's instructions. In brief, the upper chamber contained cells in control medium and the lower chamber contained IK 5, 10, 15, and 20 *μ*M, respectively. After 4 hours' incubation, the dye mixture was transferred to a 96-well microtiter plate suitable for colorimetric measurement (520 nm). Analysis was performed on four wells for each condition and each experiment was repeated three times.

### 2.9. Cell Invasion Assay

Cell invasion assay kit ECM 550 (Chemicon International Inc., Billerica, MA), containing 8-*μ*M pore size polycarbonate membranes with a thin layer of ECMatrix-likewise material, was used for invasion assays. Cells (3 × 10^5^) suspended in 300 *μ*L of serum-free medium were carefully transferred to the upper chambers of the devices and the lower chambers were filled with 500 *μ*L of serum-free medium containing IK 5, 10, 15, and 20 *μ*M, respectively. The chamber was incubated at 37°C under a humidified 5% CO_2_ atmosphere for 48 h and the number of cells that had migrated to the lower side of the filter was were counted under the microscope.

### 2.10. Cell Colony Formation Assay

Cell colonies were determined by StemTAG 96-well stem cell colony formation assay kit (Cell Biolabs, Inc. San Diego, CA, USA) according to the manufacturer's instruction. In brief, 50 *μ*L of base agar matrix layer was dispensed into each well of a 96-well plate. 50 *μ*L of cell suspension/agar matrix suspension containing 5,000 cells was dispensed into each well. After solidifying, 50 *μ*L of culture medium with IK was added into each well and the cells were incubated for 7 days. The colony formation ability was examined under a microscope and the results were presented colony number.

### 2.11. Immunofluorescence

For the localization of p65, cells were seeded onto glass coverslips in 6-well plates at a dentistry of 1 × 10^5^ cells/well. Treated cells were fixed with 4% paraformaldehyde for 30 min following permeabilization with 0.1% Triton X-100. After being washed in PBS buffer, slides were blocked with 10% normal goat serum for 1 h and then incubated with mouse monoclonal anti-human p65 antibody for 2 h at a 1 : 100 dilution in 1% normal goat serum. The slides were washed with PBS, incubated with fluorescein isothiocyanate- (FITC-) conjugated goat anti-mouse IgG at a 1 : 500 dilution in 0.5% normal goat serum for 1 hr, and counterstained for nuclei with 10 *μ*g/mL propidium iodide. Slides were imaged at ×400 magnification on a confocal microscope (Olympus, Tokyo, Japan).

### 2.12. Statistical Analysis

The statistical significance of differences between the control and treated groups was determined by a paired *t*-test or a one-way ANOVA followed by Bonferroni's multiple comparison tests. *P* values <0.05 were considered to indicate statistical significance.

## 3. Results

### 3.1. Effect of IK on HN4 and HN12 Cell Viability

**Figure 1:**
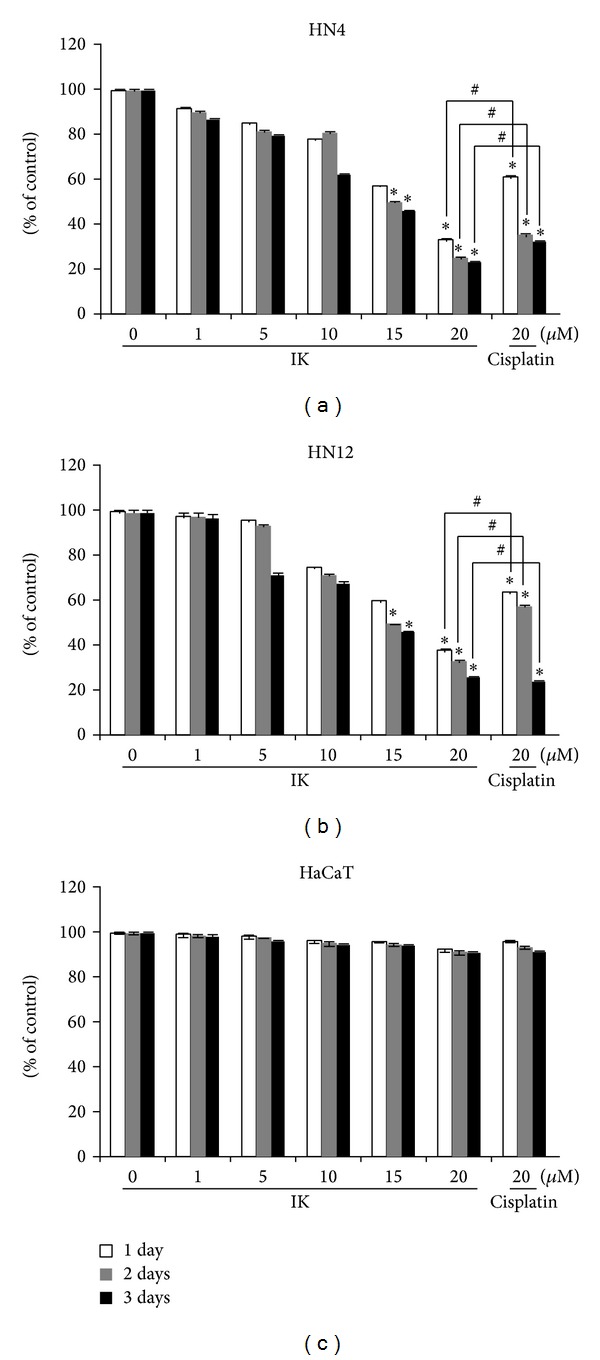
Effects of isocudraxanthone K (IK) on the viability of primary (HN4, a), metastatic oral cancer cells (HN12, b), and normal, nontumor keratinocyte cell line, HaCaT cells (c) as assessed by MTT assay. Data are the mean values of five experiments ± SD. *Significantly different from the control group (*P* < 0.05). ^**#**^Significantly different from the each group.

Inhibition of proliferation in primary (HN4) and metastatic (HN12) OSCCCs by IK is shown in Figure 1. Incubation with IK in a concentration range of 1–20 *μ*M for 72 h led to dose- and time-dependent inhibition of cell proliferation in HN4 and HN12 cells. Although the overall trend of the effects of IK on HN4 and HN12 cells was similar, IK showed higher cytotoxicity in primary HN4 cells than metastatic HN12 cells. The concentration required to inhibit cell growth by 50% (IC_50_) for HN4 (14.31 *μ*M) was lower than that of HN12 cells (14.91 *μ*M) for 3 days. For comparison, cisplatin, a drug with antineoplastic activity, was used. IK showed significantly stronger antioral cancer activity against HN4 and HN12 cells than cisplatin, when given at equitoxic doses (20 *μ*M) for 1 and 3 days of cultivation (Figures 1(a) and 1(b)). IK showed no cytotoxicity in HaCaT cells, a nontumor keratinocyte cell line (Figure 1(c)), indicating a possible therapeutic window or a selective antitumor effect.

### 3.2. Effect of IK on HN4 and HN12 Cell Apoptosis

**Figure 2:**
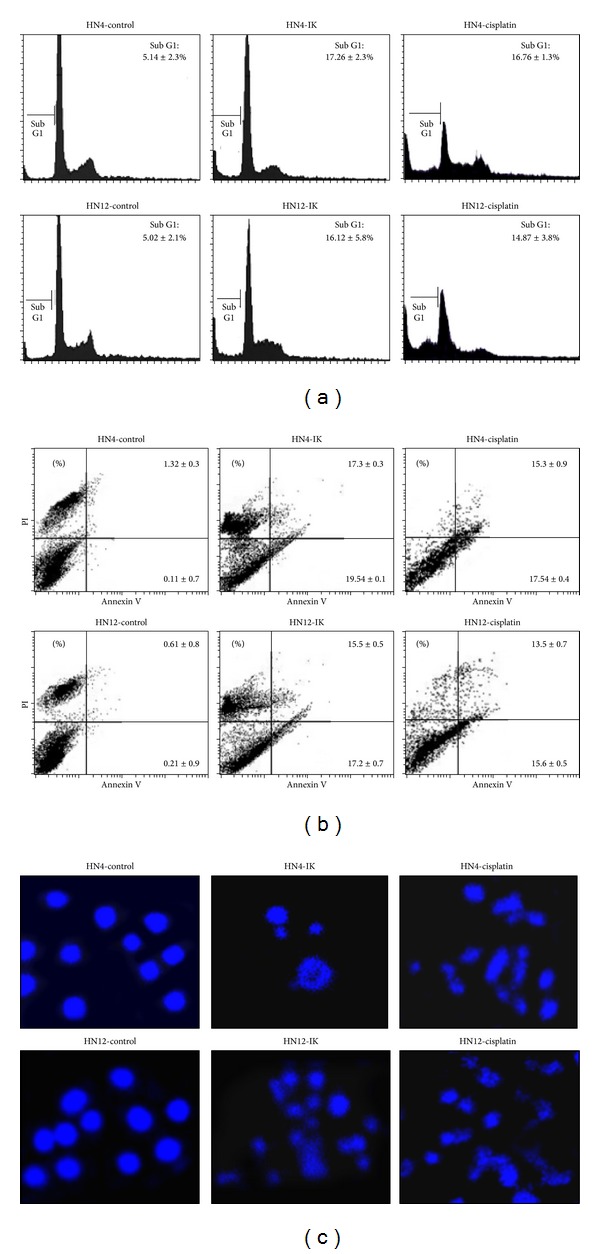
Confirmation of isocudraxanthone K- (IK-) induced apoptosis by cell cycle analysis (a), annexin V-PI flow cytometry (b), and DAPI staining (c, ×400) in HN4 and HN12 cells. Cells were incubated with 20 *μ*M IK for 2 days. The results are representative of three independent experiments.

To determine whether the growth inhibitory activity of IK was related to the induction of apoptosis, cell cycle analysis (Figure 2(a)), annexin V-FITC and propidium iodide staining (Figure 2(b)), and morphological observation of cell death using 4′,6-diamidino-2-phenylindole dihydrochloride (DAPI) staining (Figure 2(c)) were investigated. At the IC_50_ in the proliferation assays (15 *μ*M), IK induced a significant increase in the percentage of sub-G1 phase in HN4 and HN12 cells, compared with negative controls, indicating an increase in the proportion of apoptotic cells. Moreover, treatment of the HN4 and HN12 cells with IK for 2 days increased the number of early (PI^−^/AV^+^) and late apoptotic (PI^+^/AV^+^) cells. DAPI staining showed apoptotic cells with fragmented chromatin and highly condensed nuclei were also found in IK- and cisplatin-treated HN4 and HN12 cells. Together, these results suggest that IK can induce apoptosis in both primary and metastatic oral cancer cells.

### 3.3. Effect of IK on Migration, Invasion, and Colony Formation

**Figure 3:**
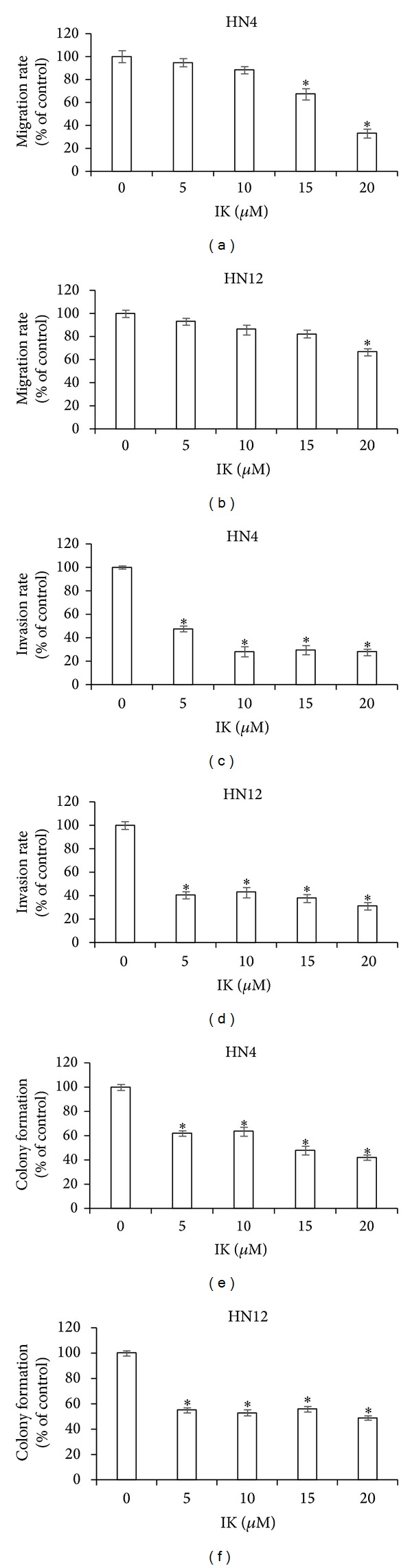
Effects of isocudraxanthone K (IK) on migration (a, b), invasion (c, d), and colony formation (e, f) in HN4 (a, c, e) and HN12 (b, d, f) cells. Transwell assay kit was employed to evaluate the migratory ability for 2 days.* In vitro* cell invasion assays were performed using the cell invasion assay kit for 2 days. Cell colony formation was measured using soft agar colony formation assay. The number of colonies was counted at 7 days after seeding the cells. Data are representative of three independent experiments (*n* = 4) and presented as % compared to nonstimulated control cells. *Statistically significant difference, compared with control, *P* < 0.05.

In order to further study the antioral cancer effects of IK, transwell migration, invasion, and soft agar assay were examined. IK effectively inhibited the migration and invasion of HN4 and HN12 cells in a dose-dependent manner compared to the untreated control cells (Figures 3(a)–3(d)). Moreover, IK decreased the number of colonies in a dose-dependent manner in both cell lines (Figures 3(e) and 3(f)).

### 3.4. Effect of IK on Apoptosis-Regulating Proteins in HN4 and HN12 Cell

**Figure 4:**
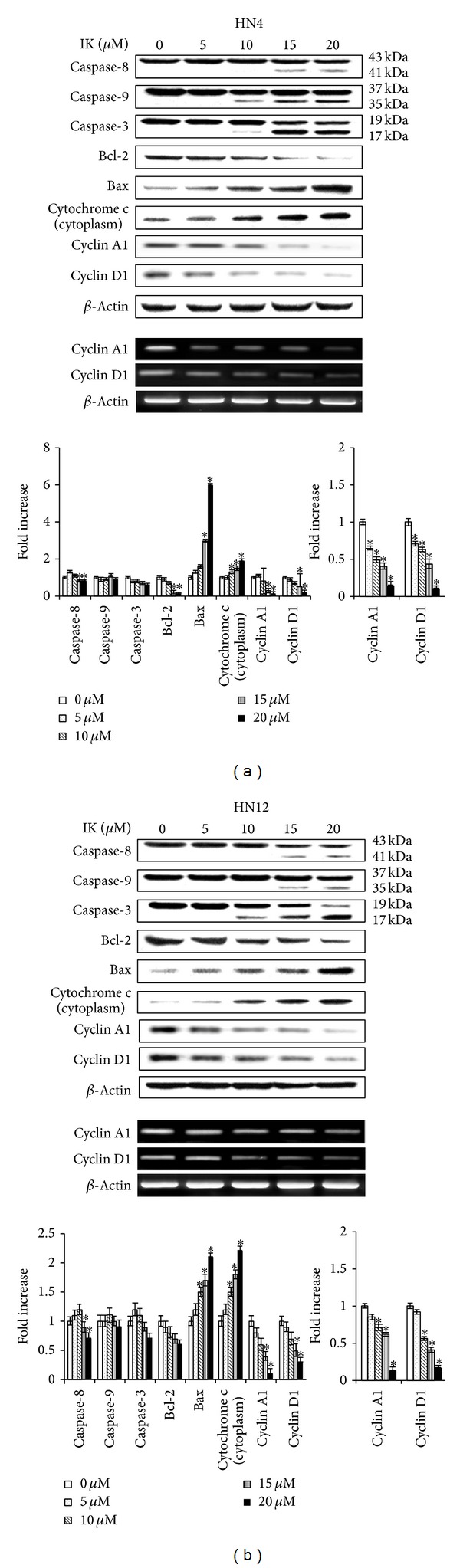
Effects of isocudraxanthone K (IK) on expression of apoptosis regulatory proteins in HN4 (a) and HN12 (b) cells. Cells were cultured with the indicated dose of IK for 2 days. The histogram shows the quantification of mRNA or protein expression by densitometry and is presented as fold increases compared to nonstimulated control cells. The results are representative of three independent experiments. *Statistically significant difference, compared with control, *P* < 0.05.

To examine the possible mechanism of IK-induced cell cycle arrest during the sub-G_1_ phase, the levels of cyclin D1 and cyclin A1 were assessed in HN4 and HN12 cells exposed to IK (Figures 4(a) and 4(b)). The results revealed that IK stimulation in HN4 and HN12 cells significantly reduced the level of cyclin D1 and cyclin A1 mRNA and protein in a dose-dependent manner.

To clarify the potential targets of IK in apoptosis-inducing pathways in human OSCCCs, we investigated the involvement IK in a caspase cascade (Figure 4). Initiator caspases, such as caspase-8 and caspase-9, and effector caspases, such as caspase-3, were activated by IK in primary and metastatic OSCCCs with 48 h of exposure, in a dose-dependent manner. Because IK activated both initiator and executioner caspases, we next examined the involvement of Bcl-2 family proteins and of cytochrome c release from mitochondria in IK-treated OSCCCs by Western blotting (Figure 4). Treatment of OSCCCs with IK resulted in a dose-dependent reduction in the levels of the antiapoptotic protein Bcl-2 with a concomitant increase in the levels of the proapoptotic protein Bax, compared with the control. Moreover, cytochrome c was found to be released from mitochondria into the cytosol in a concentration-dependent manner.

### 3.5. Effect of IK on Akt, MAPK, and NF-*κ*B Activation

**Figure 5:**
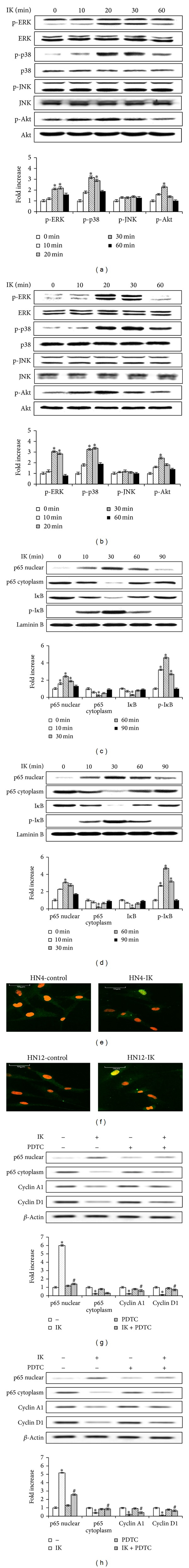
Effects of isocudraxanthone K (IK) on phosphorylation of MAPK and Akt (a, b) and activation of NF-*κ*B (c, d, e, f) in HN4 (a, c, e) and HN12 (b, d, f) cells. Effects of NF-*κ*B inhibitor PDTC on IK-induced cyclin D1 and cyclin A1 expression (g, h) in HN4 (g) and HN12 (h) cells. Cells were cultured without or with 20 *μ*M IK for the indicated times (a–d) or 30 min (e, f). Cells were pretreated with 1 mM of PDTC for 1 h and then posttreated with IK 20 M (g, h). Signaling pathways were assessed via Western blot (a–d, g, h) and immunofluorescence staining (e, f). Results are representative of three independent experiments. The histogram shows the quantification of gene expression by densitometry and is presented as fold increases compared to nonstimulated control cells. *Statistically significant difference, compared with control, *P* < 0.05. ^#^Statistically significant difference, compared with IK group, *P* < 0.05.

Akt is known to regulate survival and apoptosis by phosphorylation of a number of other downstream signalling proteins such as MAPK [8, 35]. The MAPK family comprises three major subfamilies:  p38, extracellular-signal regulated kinases (ERK), c-Jun N-terminal kinases (JNK). Since Akt, MAPK families, and NF-*κ*B influence cell survival and apoptosis [8, 36], the effect of IK (20 *μ*M) on the levels of phosphorylation and activation of these cellular signaling pathways were assessed. IK elicits a rapid phosphorylation of p38 MAPK and ERK with a peak at 20 or 30 minutes in HN4 and HN12 cells, but not JNK (Figures 5(a) and 5(b)). In contrast, IK did not affect total p38, ERK, or JNK levels in HN4 and HN12 cells. As shown in Figures 5(a) and 5(b), IK treatment markedly increased the phosphorylation of Akt at 20 min.

MAPKs can participate in the regulation of nuclear factor *κ*B (NF-*κ*B) activation via phosphorylation and degradation of I-*κ*B*α* and nuclear translocation of NF-*κ*B p65 [36]. The NF-*κ*B activities of OSCCCs treated with IK were measured by examining the nuclear translocation of NF-*κ*B subunit p65 and phosphorylation and degradation of I-*κ*B*α*. Following IK treatment, increased levels of phosphorylated and degraded I-*κ*B*α* were noted within 20 min and maximal levels were noted at 30 min. IK increased the nuclear translocation of p65 subunit of NF-*κ*B after 20 min of treatment and remained enhanced up to 90 min (Figures 5(c) and 5(d)). To ascertain whether p65 nuclear translocation following exposure to IK occurred, we used an immunofluorescence assay to confirm nuclear localization. Treatment with IK caused a marked shift in the fluorescence signal into the nucleus, reflecting the IK-mediated nuclear translocation of the p65 (Figures 5(e) and 5(f)). To better understand the role of NF-*κ*B, the effects of NF-*κ*B inhibition on expression of target genes of NF-*κ*B such as cyclin D1 and cyclin A1 were examined. The results showed that NF-*κ*B inhibitor, pyrrolidine dithiocarbamate (PDTC), pretreatment recovered IK-suppressed cyclin D1 and cyclin A1 expression (Figures 5(g) and 5(h)).

### 3.6. Involvement of the HIF-1*α* Pathway in IK-Induced Apoptosis

**Figure 6:**
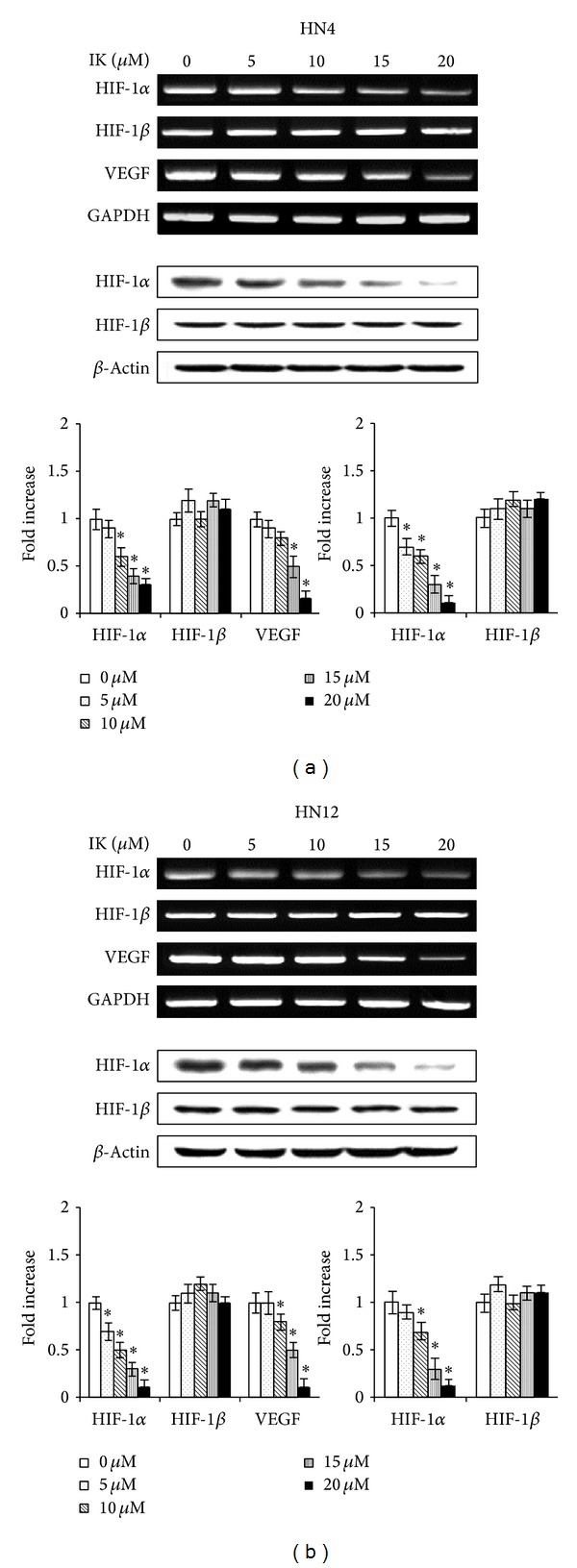
Effects of isocudraxanthone K on expression of HIF-1*α* and VEGF in HN4 (a) and HN12 (b) cells. Cells were cultured without or with various concentrations of IK for 2 days. mRNA and protein levels were assessed by RT-PCR and Western blot analysis, respectively. The results are representative of three independent experiments. The histogram shows the quantification of gene expression by densitometry and is presented as fold increases compared to nonstimulated control cells. *Statistically significant difference, compared with control, *P* < 0.05.

**Figure 7:**
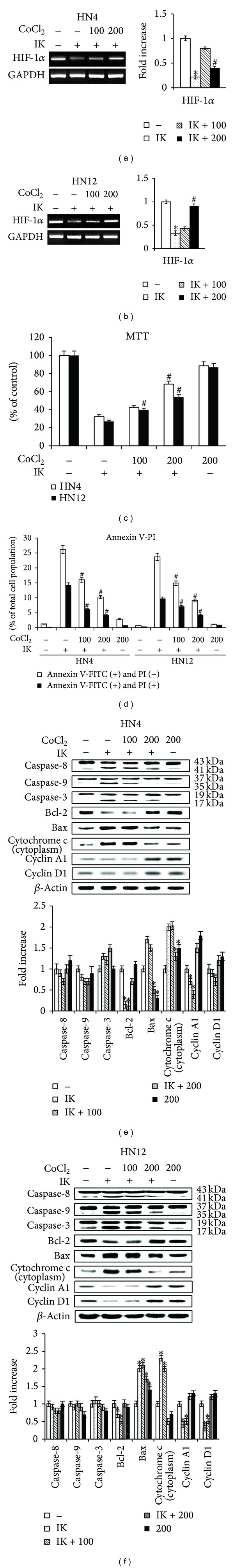
Effects of HIF-1*α* induction by cobalt chloride (CoCl_2_) on isocudraxanthone K (IK)-induced HIF-1*α* expression (a, b), cytotoxicity (c), early (PI^−^/AV^+^) and late apoptotic (PI^+^/AV^+^) cells (d), and apoptosis-regulated proteins (e, f) in HN4 (a, c, d, e) and HN12 (b, c, d, f) cells. Cells were pretreated with 100 or 200 *μ*M CoCl_2_ for 6 h and posttreated with 20 *μ*M for 2 days. The histogram shows the quantification of gene expression by densitometry and is presented as fold increases compared to nonstimulated control cells. *Statistically significant difference, compared with control, *P* < 0.05. ^#^Statistically significant difference versus IK, *P* < 0.05.

To determine the possible mechanisms of IK-induced apoptosis in OSCCCs, we also analyzed whether IK affected the expression of HIF-1*α* and its target gene, VEGF. IK reduced HIF-1*α* mRNA and protein levels in a concentration-dependent manner (Figures 6(a) and 6(b)). However, HIF-1*β* mRNA and protein levels were unaffected. IK also reduced VEGF mRNA expression, dose dependently (Figure 6). To identify whether HIF-1*α* inhibition was involved in the growth-inhibiting and apoptosis-inducing effects of IK, OSCCCs were pretreated for HIF-1*α* induction with cobalt chloride (CoCl_2_). CoCl_2_ dose dependently recovered IK-induced HIF-1*α* mRNA levels in HN4 and HN12 cells (Figure 7(a)). Additionally, IK-induced cytotoxicity and apoptosis in HN4 and HN12 cells were reversed dose dependently by pretreatment with CoCl_2_ (Figures 7(b) and 7(c)).

Finally, we examined whether HIF-1*α* modulated the mitochondrial and death receptor signaling pathways during IK-induced apoptosis in OSCCCs. Our results showed that CoCl_2_ treatment blocked the induction of caspase-8, caspase-9, caspase-3, Bax, and cytochrome c expression by IK but reversed the expression of Bcl-2 in HN4 and HN12 cells (Figures 7(e) and 7(f)).

## 4. Discussion

In a search for natural product-derived inhibitors of HIF-1*α*, we investigated IK, a new compound isolated from* C. tricuspidata* roots. We used OSCCCs to assess its antiproliferative activity and its mechanism of action was examined by studying the key markers involved.

In the present study, we found that IK resulted in significant growth inhibition of primary and metastatic OSCCs. Furthermore, 20 *μ*M IK appeared to be a more potent inhibitor of cell viability in primary and metastatic OSCCCs than the same dose of cisplatin, suggesting that IK may be a drug lead from a plant source. These growth-inhibitory properties of IK are similar to our previous report that another compound, cudraflavone B, also isolated from* C. tricuspidata* roots, is cytotoxic in OSCCCs [32]. Moreover, we demonstrated that IK induced OSCC cell death by triggering apoptosis due to the presence of several apoptotic characteristics, including sub-G_1_ phase accumulation, increase in annexin^+^/PI^+^ cells, and DNA fragmentation.

A key mechanism involved in the action of many anticancer drugs is activation of the mitochondrial apoptotic pathway. Initiator caspases, typically caspase-8 and caspase-9, are activated by two alternate pathways. The first involves cell-death-receptor-mediated apoptosis through caspase-8 [37]. The second involves mitochondria-mediated apoptosis through caspase-9. The key element in the pathway is the efflux of cytochrome c from mitochondria to the cytosol. In the cytosol, cytochrome c, together with Apaf-1, activates caspase-9, which then activates caspase-3 [38]. In both pathways, the initiator caspase cleaves and activates downstream effector caspases, such as caspase-3 [38]. In our study, treatment with IK stimulated caspase-8, caspase-9, and caspase-3 activation and the cytosolic release of cytochrome c. In addition, IK induced the upregulation of Bax, but downregulation of Bcl-2. Our results indeed suggest that the mitochondrial pathway and the death receptor signaling pathway were both involved in apoptosis induced by IK in OSCCCs.

MAPK, PI3K/Akt, and NF-*κ*B are important in regulating cell apoptosis and proliferation. Akt, an important and probably essential downstream component of PI3K-mediated oncogenic signaling, provides a critical cell survival signal for tumor progression by phosphorylating proteins involved in cell cycle regulation and proapoptotic factors [8, 39]. The MAPK signaling pathway has been shown to be activated in response to certain chemotherapeutic drugs [40]. In the present study, the roles of Akt, MAPK, and its downstream transcription factor NF-*κ*B in regulating IK-induced apoptosis were examined in primary and metastatic OSCCs. Our results indicate that IK leading to tumor cell apoptosis is attributable to the activation of the Akt, ERK, and p38 pathway because the levels of phosphorylated Akt, ERK, and p38 were gradually increased after exposure to IK. This result is similar to previous reports that sappanchalcone [12] and mollugin [14] exerted anticancer effects through p38, ERK, and JNK activation in OSCCCs. Furthermore, selenocysteine treatment triggered the activation of JNK, p38 MAPK, ERK, and Akt in breast cancer cells [41].

NF-*κ*B is a nuclear target of MAPK signaling pathways. In many instances, phytochemicals such as curcumin, capsaicin, gingerol, flavopiridol, genistein, and diosgenin have been shown to suppress NF-*κ*B during apoptosis [42]. Thus, it is generally accepted that inhibition of NF-*κ*B was suggested to be a useful strategy for cancer therapy [36]. However, several studies demonstrated that chemotherapeutic agents, such as etoposide, CPT-11, adriamycin, vincristine, and taxol, can induce NF-*κ*B activation [43–45]. The present study showed that IK induced NF-*κ*B activation in OSCCs, as evidenced by increased NF-*κ*B p65 nuclear translocation, phosphorylated I-*κ*B*α* level, and subsequent proteolytic degradation of I-*κ*B*α*. This finding suggests that NF-*κ*B activation provides a cell survival signal following IK treatment. Our results were similar to recent studies showing NF-*κ*B activation by sappanchalcone [12], isoliquiritigenin 2′-methyl ether (ILME) [13], and cudraflavone B [32] in OSCCCs. The reason for these controversial findings in the role of NF-*κ*B activation in apoptosis remains to be determined but may relate to biological differences in cells types and different stimuli. To further elucidate the effect of NF-*κ*B activation on IK-induced apoptosis of OSCCCs, PDTC, a potent NF-*κ*B inhibitor, was used. Our results showed that the combination of PDTC with IK reversed the IK-induced downregulation of cyclin D1 and cyclin A1 expression. These data support NF-*κ*B activation may be responsible for apoptosis in OSCCCs response to the proapoptotic effect of IK. In addition, our observations suggest that IK-induced p38, ERK, Akt, and NF-*κ*B activation may lead to the expression of downstream target genes including caspases, Bcl-2, Bax, cytochrome c, and cyclins.

HIF-1*α* is an important transcriptional regulator that controls the transcription of the vascular endothelial growth factor (VEGF) gene [15, 16]. Whether HIF-1*α* can promote tumor cell apoptosis or antiapoptosis has been controversial. Overexpressed HIF-1*α* can promote apoptosis by activating Bcl-2 and Bcl-Xl or enhancing the stability of p53 [46]. In contrast, HIF-1*α* can upregulate VEGF and GLUT1, making tumor cells resistant to apoptosis, so inhibition of HIF-1*α* would promote apoptosis [47]. Furthermore, tumor reduction was found in nude mice implanted with human prostate cancer cells treated with the HIF-1*α* inhibitor EZN-2968 [48]. Our results show that IK downregulated HIF-1*α* and VEGF expression in a dose-dependent manner in HN4 and HN12 cells. We found that growth inhibition and apoptosis of OSCCs by IK was reversed in the presence of a HIF-1*α* activator, CoCl_2_. These results are consistent with previous data that CoCl_2_ attenuated tert-butyl hydroperoxide-induced apoptotic death in the hepatoma cell line HepG2 [49] and reduced cisplatin-induced apoptosis in human lung adenocarcinoma A549 cells [46]. In the present study, IK induced caspase-8, caspase-9, caspase-3, Bax, and cytochrome c expression, while downregulation of Bcl-2, cyclin D1, and cyclin A1 levels was reversed by CoCl_2_ in a dose-dependent manner. These results suggest that the growth-inhibiting and apoptosis-inducing effects of IK are HIF-1*α* mediated in OSCCCs.

In conclusion, we demonstrated for the first time that isocudraxanthone K, a novel, natural compound inhibits proliferation and induces apoptosis in OSCCCs through mitochondria/death receptor, MAPK, NF-*κ*B, and HIF-1*α* signaling pathways. Thus, isocudraxanthone K may be a promising candidate for oral squamous cell carcinoma therapy.

## Abbreviations

MAPK: Mitogen-activated protein kinase
NF-*κ*B: Nuclear factor-*κ*B
OSCCCs: Oral squamous cell carcinoma cells
HIF-1*α*: Hypoxia-inducible factor-1*α*.
