# Erratum to “Isocudraxanthone K Induces Growth Inhibition and Apoptosis in Oral Cancer Cells via Hypoxia Inducible Factor-1*α*”

**DOI:** 10.1155/2020/5464068

**Published:** 2020-10-15

**Authors:** Mee-Ran Shin, Hwa-Jeong Lee, Soo-Kyung Kang, Q-Schick Auh, Young-Man Lee, Youn-Chul Kim, Eun-Cheol Kim

**Affiliations:** ^1^Department of Prosthodontics, Dongtan Sacred Heart Hospital, Hallym University, Dongtan, Republic of Korea; ^2^Department of Oral and Maxillofacial Pathology, and Research Center for Tooth and Periodontal Regeneration (MRC), School of Dentistry, Kyung Hee University, 1 Heogi-dong, Dongdaemun-gu, Seoul 130-701, Republic of Korea; ^3^Department of Oral Medicine, School of Dentistry, Kyung Hee University, Seoul, Republic of Korea; ^4^College of Pharmacy, Wonkwang University, Iksan 570-749, Republic of Korea

In the article titled “Isocudraxanthone K Induces Growth Inhibition and Apoptosis in Oral Cancer Cells via Hypoxia Inducible Factor-1*α*” [1], there was an error in Figure 5(h) where the panels for HN12 cells were identical to those in Figure 5(g) (HN4 cells). This error occurred in the production process. The figure should be corrected as follows:

## Figures and Tables

**Figure 1:**
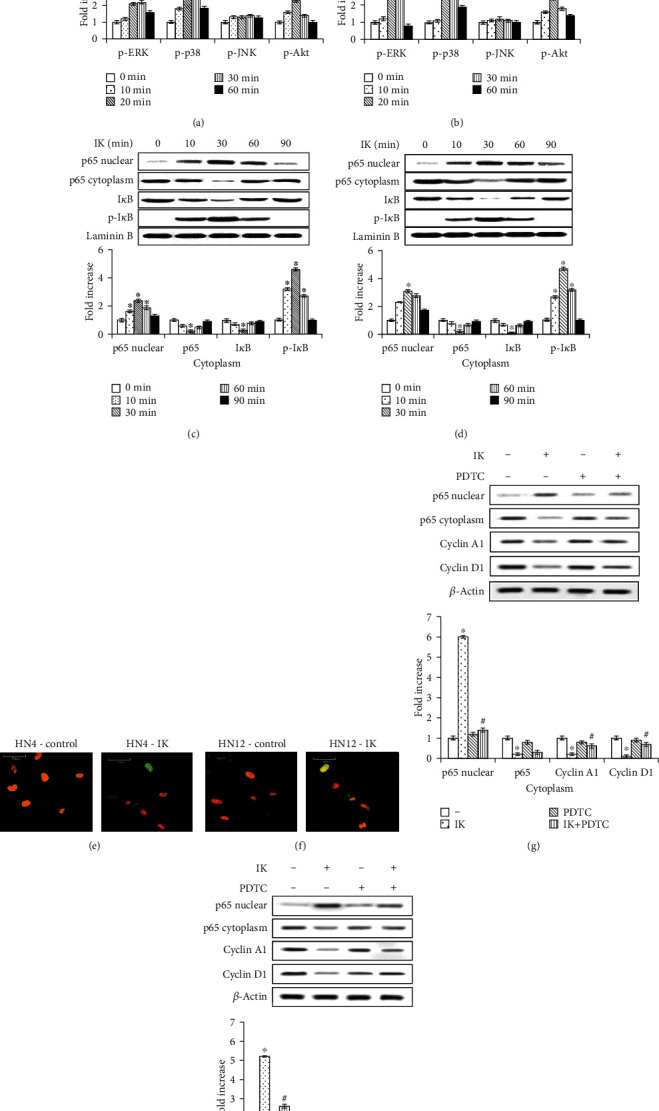
Effects of isocudraxanthone K (IK) on phosphorylation of MAPK and Akt (a, b) and activation of NF-*κ*B (c–f) in HN4 (a, c, e) and HN12 (b, d, f) cells. Effects of NF-*κ*B inhibitor PDTC on IK-induced cyclin D1 and cyclin A1 expression (g, h) in HN4 (g) and HN12 (h) cells. Cells were cultured without or with 20 *μ*M IK for the indicated times (a–d) or 30 min (e, f). Cells were pretreated with 1 mM of PDTC for 1 h and then posttreated with IK 20 M (g, h). Signaling pathways were assessed via Western blot (a–d, g, h) and immunofluorescence staining (e, f). Results are representative of three independent experiments. The histogram shows the quantification of gene expression by densitometry and is presented as fold increases compared to nonstimulated control cells. ^∗^Statistically significant difference, compared with control, *P* < 0.05. ^#^Statistically significant difference, compared with IK group, *P* < 0.05.
